# Pseudo-random Path Generation Algorithms and Strategies for the Surface Quality Improvement of Optical Aspherical Components

**DOI:** 10.3390/ma13051216

**Published:** 2020-03-08

**Authors:** Jun Zha, Hangcheng Zhang, Yipeng Li, Yaolong Chen

**Affiliations:** 1 School of Mechanical Engineering, Xi’an Jiaotong University, Xi’an 710049, China; chenghangz@foxmail.com (H.Z.); liyipeng@mail.xjtu.edu.cn (Y.L.); chenzwei@mail.xjtu.edu.cn (Y.C.); 2 Shenzhen Research School, Xi’an Jiaotong University, Hi-Tech Zone, Shenzhen 518057, China; 3 Xi’an Jiaotong University Suzhou Academy, 99 Renai Road, Suzhou 215123, China

**Keywords:** aspherical surface, surface roughness, surface waviness, polishing path generation

## Abstract

This study proposes two path generation algorithms to diminish the superposition of the convolution effect on the polishing path in computer-controlled optical surfacing. According to the polishing of aluminum-alloy based hyperboloid optical components, different proportions of polishing agents were blended. Then, the surface roughness of the optical components were determined through a validation experiment of the algorithms. Furthermore, the relationship between surface roughness and the polishing agent concentration, and the compensation strategies for surface roughness were analyzed. The results show that the two algorithms effectively compensated for surface waviness. The findings support the strategies for improving the surface quality of optical components with aspherical surfaces.

## 1. Introduction

With the advancement of research in the fields of high-energy physics and microscopic observation [1], the demand for optical components with aspherical surfaces, which provide customizable designs with excellent performance, as compared to all-spherical solutions, is increasing [2]. The optical performances of such optical components are affected by several factors.

By expressing the surface shape in the Fourier series, these factors can primarily be divided into three types [3]: 1) surface roughness, High Spatial Frequency Range with wavelengths < 0.12 mm; 2) surface waviness, Middle Spatial Frequency (MSF) Range with wavelengths between 0.12 and 33 mm; and 3) surface profile error, Low Spatial Frequency Range with wavelengths > 33 mm.

Among them, the surface waviness error of key optical components leads to an obvious peak intensity, which might damage the optical components [4]. In addition, the surface roughness of optical components affects imaging clarity, particularly around focal points.

Power Spectral Density (PSD) character curves have been proposed to evaluate the errors of different frequency ranges [5]. Surface profile error and surface roughness are primarily compensated for by modifying the dwell time of optimization [6,7] and the convolution of the removal function, which are strongly related to the polishing strategy [8,9]; even the most difficult processing issues of edge mis-figure can be controlled [10,11]. Researchers have conducted comprehensive studies on this topic, and suggested that the surface profile error can be diminished through small-tool polishing [12], magneto-rheological finishing [13,14,15], and stressed lap polishing [16]. Tam et al. [17,18] presented a comparison of Peano-like paths and Hilbert-like paths of small-tool polishing. Li et al. [19] utilized the fractal Tool-Path planning strategy to diminish the roughness error. Dong et al. generated a random fractal-like tool path that possesses multi-directionality during multiple polishing iterations with better performances in terms of restraining the polishing induced surface ripples of round-shaped flat-fused silica [20]. Schinhaerl [21] proposed the influence function of computer-controlled optical surfacing (CCOS) to simulate the polishing process. Dunn et al. [22] used Pseudo-random tool paths and achieved an obvious improvement in the performance of a bonnet-polishing machine. Circular pseudo-random paths have the advantages of the ability to suppress velocity changes of the polishing tool and thus restrict the degradation of surface waviness, especially polishing runs at fast speed [23]. Wang et al. proposed a unicursal random maze tool path algorithm and verified the effectiveness of restraining the mid-spatial frequency error, in comparison to the Hilbert path [24]. Zhao et al. investigated a six-directional pseudorandom consecutive unicursal polishing path and validated the processed work piece, which had a significantly lower MSF error [25]. Some other polishing paths, such as the ‘hyper-crossing’ tool-path, which crosses itself, can be used to minimize MSFs in Computer Numerical Control (CNC) precession bonnet-polishing [26]. The planning of the tool path is beneficial for minimizing dimensional errors [27], which determine the focusing properties and optical transfer function of optical components that directly affect the surface profile. Some other method, like the work on spherical lens in copper were approached by using bio-machining [28]. Also, custom-shaped milling tools [29], barrel end mills [30,31] and conical milling tools [32] are beneficial for the free-form machining with high accuracy and high efficiency requirement. In addition, the roughness requirement in complex surface test part could be met by forming tools in high-speed finishing [33]. In practice, energy transfer in the low frequency range will decline rapidly in an unsuitable optical surface, and this part of the energy will accumulate in the interior of the optical mirror, which may lead to accidents. To compensate for the surface profile errors in the middle spatial frequency range, a special polishing path is often designed with two requirements: (1) to avoid the negative effects of the compensation in the low frequency range, and (2) to ensure the surface quality consistency of the whole polished surface.

This paper presents a compensation method for the surface quality improvement of optical aspherical components. Experiments, based on CCOS, were conducted to determine the strategy for improving the surface quality of optical components. Path generation algorithms are also proposed to change the direction and interval of the path. The effectiveness of the algorithms was verified through a validation experiment. In addition, different proportions of polishing agents were blended and corresponding polishing experiments were carried out. Surface roughness data after polishing were collected, and the relationship between the surface roughness and polishing agent concentration were determined, and the strategy to compensate for surface roughness was analyzed. The proposed path direction changing algorithm and path interval changing algorithm possess the characteristics of resolving the surface waviness caused by the existence of the large parallel path and convolution effects between path intervals, respectively.

## 2. Generation Mechanism of Surface Waviness Error

In CCOS, machine waviness is generated on aspherical surfaces, which is known as the surface waviness error. To develop a suppressing method, the error generation mechanism should first be clarified.

The surface shape error of an aspherical surface can effectively be tracked and removed through a CCOS method based on small-tool polishing. Compared to the normal method, this method has a stronger machining ability and can process the more complex surface profiles of optical components with aspherical surfaces. However, it would also produce a new processing waviness error on aspherical surfaces, which is mainly affected by the following factors [22]: (1) high frequency range error of the initial profile of the aspherical surface; (2) unstable time-varying characteristic of the removal function during machining; and (3) convolution effect of the CCOS polishing process. The initial profile error can be avoided by applying some special tools during the process of the initial roughing. To stabilize the removal function, pre-testing certain conditions of the removal function and controlling the polishing process in real time would be effective. The convolution effect is the main reason for the surface waviness error of free surfaces, which is mainly caused by the superposition of removal functions between different polishing paths.

**Figure 1:**
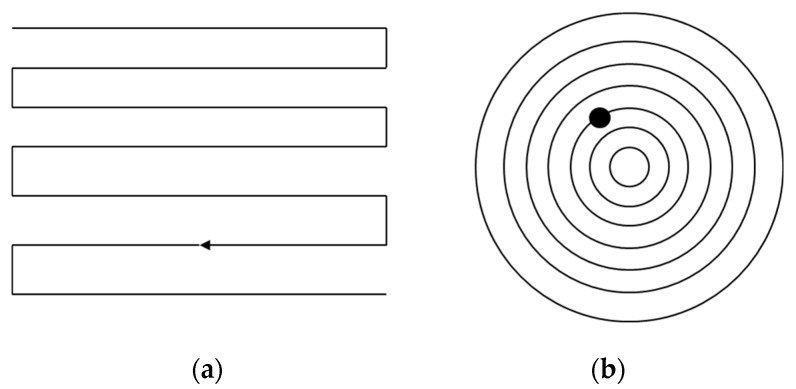
Conventional polishing paths based on CNC processing: (**a**) raster polishing path and (**b**) concentric circle path.

At present, the conventional polishing path based on CNC small-tool polishing adopts the same method as the milling and polishing of optical components at the same stage. Generally, the raster processing path or concentric circle processing path is adopted, as shown in Figure 1. The raster polishing path produces a transverse waviness error on the surface, whereas the concentric circle path produces a circular waviness error.

**Figure 2:**
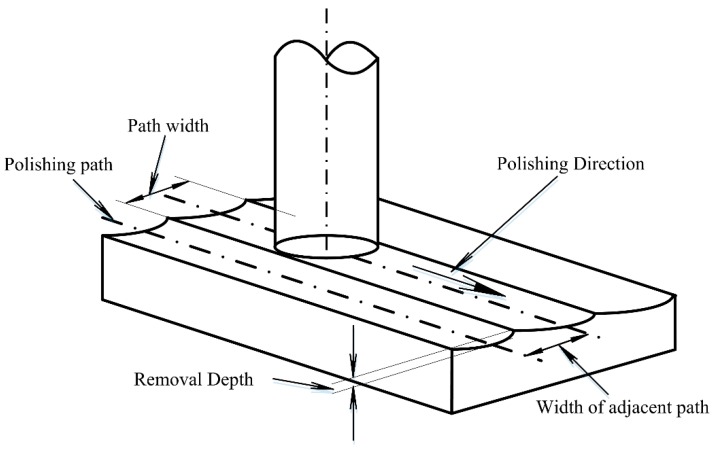
Schematic diagram of the surface waviness error generation mechanism.

The mechanism for this error is, during the polishing process, the polishing tool will feed along the polishing path. Next, in the normal direction of the polishing path, the feed is discontinuous. Therefore, there will be intervals. Moreover, the removal function of the polishing also has a removal effect on the area outside the polishing path. Under the combined action of the convolution effect and discontinuity of the path, the waviness polishing errors will appear, as shown in Figure 2. The spacing of the polishing path is assumed to be the width of the removal function. Then, waviness errors would appear in the normal direction of the path.

The function of the surface waviness error is shown in Equation (1):

(1) $$E\left(x_{i},y_{i}\right)=H\left(x_{i},y_{i}\right)-\overset{n}{\underset{j=1}{\sum }}Z\left(x_{i},y_{j}\right)⊗D\left(x_{i},y_{j}\right)$$

where *E*(*x_i_*,*y_i_*) represents the residual error after polishing, and n represents all the positions in the path that will be convolution-coherent with (*x_i_*,*y_i_*). The ⊗ means convolution calculation, *H*(*x_i_,y_i_*) is the actual material removal rate, *D*(*x_i_,y_i_*) is dwell time, and *Z*(*x_i_,y_i_*) is removal function. Theoretically, when the width of the removal function is infinitely small, the function can be expressed by the impulse function, and this error will not appear in the correction of the surface profile. However, in the actual polishing process, it is impossible to make the width of the removal function satisfy ideal conditions. Therefore, the problem needs to be addressed with other approaches. To solve this problem, this study proposes a method of modifying the polishing path.

## 3. Polishing Path Generation Algorithm

In order to avoid the superposition of the convolution effect on the polishing path, two strategies can be adopted:

(1) Changing the path formation direction to diminish parallel or concentric path lines on the surface of the optical component.
(2) Changing the path interval; a uniform feed of conventional paths is added with uncertainty to suppress the appearance of the surface waviness error.

### 3.1. Path Direction Changing Algorithm

**Figure 3:**
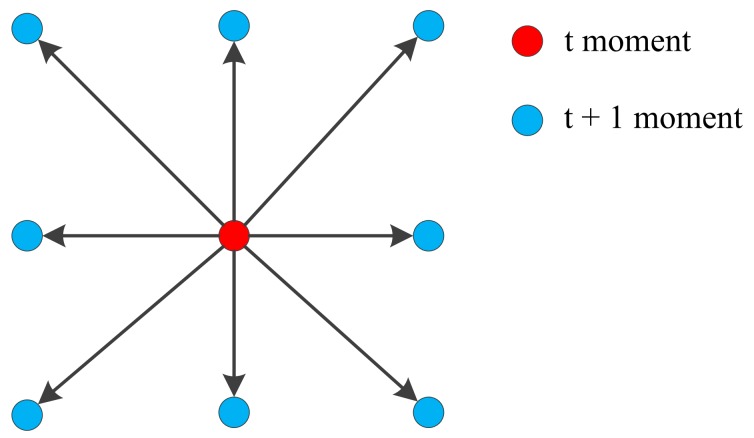
Demonstration of randomly selected path points.

Parallel path lines can be avoided by introducing uncertainty in the path planning. When the polishing path interval is set to 1 mm, as is the caser for the conventional path, the polishing head stays at the position of (x,y) at a certain time t. Then, during the next time t + 1, the polishing head would be at the position of (x + 1,y) or (x − 1,y). After adding the uncertainty of the path generation, the position of the polishing head at the next moment (t + 1) is randomly selected around the position of the t moment, as shown in Figure 3. Any one of the blue point may be selected after the red point, and they are stored as candidate sets in the path direction-changing algorithm.

The point selection method proposed above is a random point method, but in the path selection, certain constraints need to be added to the selection of the next polishing point, making it degenerate into a pseudo-random point selection. The constraints are as follows:

(1) All dwell points can only be traversed once,
(2) The dwell point (x, y) of the path cannot intersect the existing path,
(3) The path cannot exceed the boundary of the actual polishing area.

**Figure 4:**
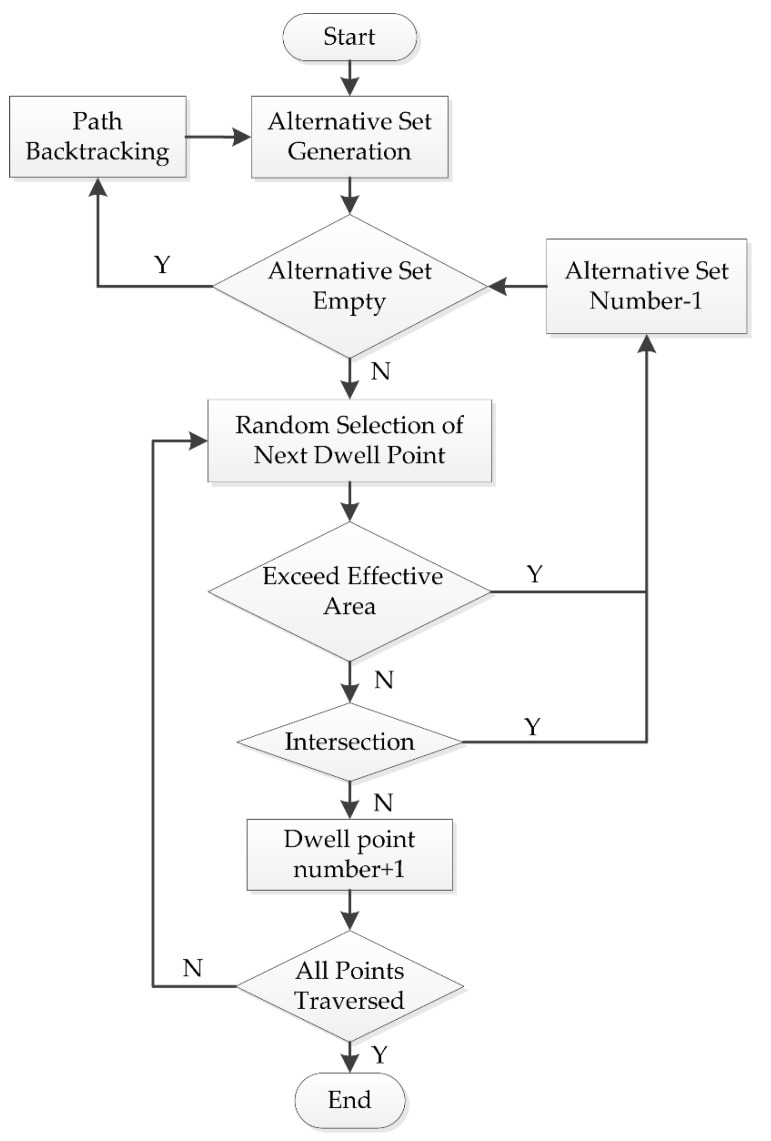
Flow chart of the changing path direction algorithm.

According to the analysis above, the path generation algorithm can be obtained, as shown in Figure 4.

To generate the selection set, the points around the current position need to be preliminarily investigated to determine whether they have been traversed or not. Then, the points not traversed should be included in the selection set. If the randomly selected point R does not meet the requirements, the size of the selection sets will be reduced by one, and new points will continue to be randomly selected from the remainder of the selection set.

**Figure 5:**
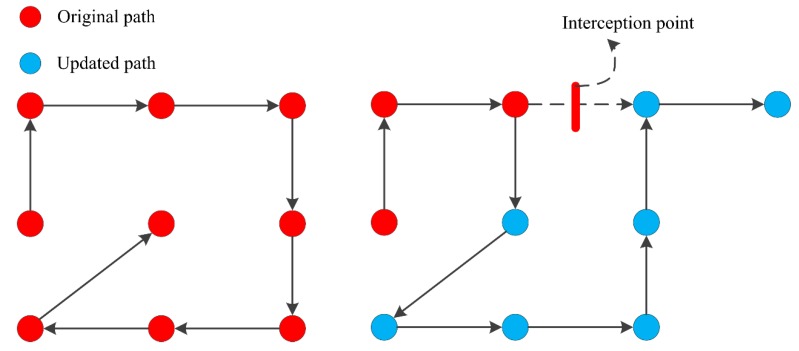
Schematic diagram of the path backtracking strategy.

If the selection set proves to be undesirable, which means that the points around the position do not satisfy the constraints, the path would become an infinite loop. Then, the flow will enter the “path backtracking” step. The strategy of the path backtracking step is to intercept the path from a point R(t’) around the current point R(t), directly connect the point R(t’) to the current point R(t), and then backtrack to the previous point of the interception point to form a new path. The current position of the new path is R(t’ + 1), which is the next point of the interception point in the original path. This allows for the reprogramming of the original path, while maintaining most of it, and updating of the selection set. The strategy is shown in Figure 5.

**Figure 6:**
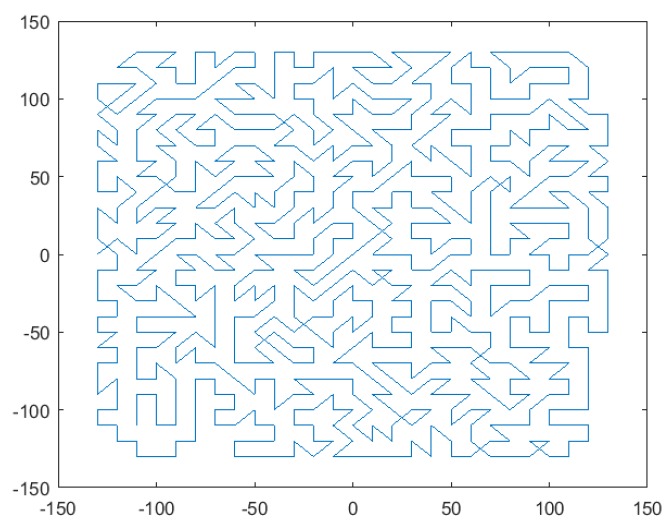
Results of the pseudo-random path under the polishing path width of 10 mm.

Under the premise of ensuring the random generation of the path direction, the above method adds some constraints to enhance the randomness and the astatic feature of the polishing path effectively. In this manner, the coherent error can be reduced significantly. Figure 6 shows the results of random paths for 10 mm steps with the above strategy.

### 3.2. Path Interval Changing Algorithm

**Figure 7:**
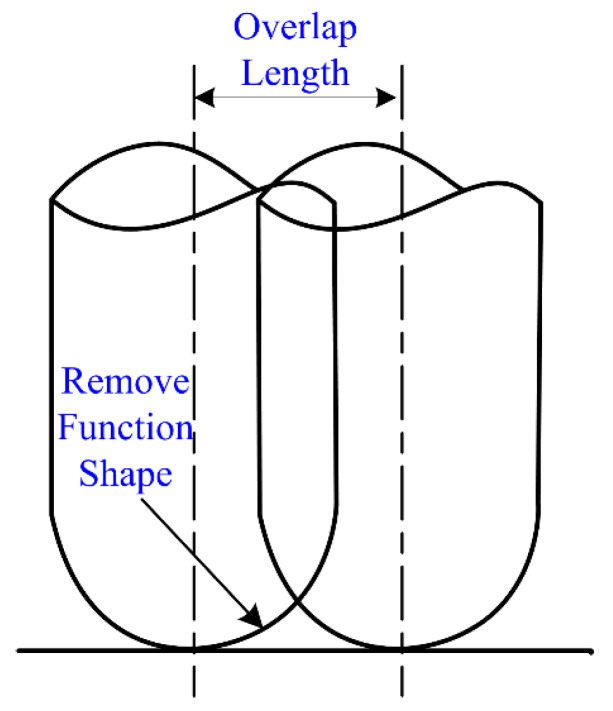
Schematic diagram of the overlapping path.

On the basis of the path lines, the spacing between parallel path lines was modified to obtain the overlap rate between the intervals (Figure 7), which could compensate for the removal amount missing in the interval of the Gaussian removal function and diminish the surface waviness error.

The overlap is defined as Equation (2):

(2) $$\mathrm{overlap}=\frac{r^{\prime }}{2\times r}$$

where *r*’ represents the overlapping length of the adjacent paths, and *r* represents the diameter of the removal function. In order to evaluate the filling effect of the path overlap, standard deviation was selected, and the calculation formula is expressed as Equation (3):

(3) $$\sigma =\sqrt{\frac{1}{N}\overset{N}{\underset{i}{\sum }}{\left(X_{i}-\mu \right)}^{2}}$$

where *N* is the path width (as shown in Figure 2), *X* is the mass removal rate at every point, and *μ* is the average of *X*. The standard deviation represents the degree of data dispersion, and the higher the degree of dispersion, the weaker the filling effect. Hence, the overlap rate corresponding to the minimum standard deviation should be adopted.

The parameters of the removal function of a planetary polishing tool used in this study are as follows: rotation rate ratio n = −1 and eccentricity ratio e = 0.8. The standard deviation of the removal function with different overlap rates was calculated, and the results are shown in Table 1. According to the results, the filling effect is the most significant when the overlap rate is 40%, and continued increase of the overlap rate will reduce the filling effect.

**Figure 8:**
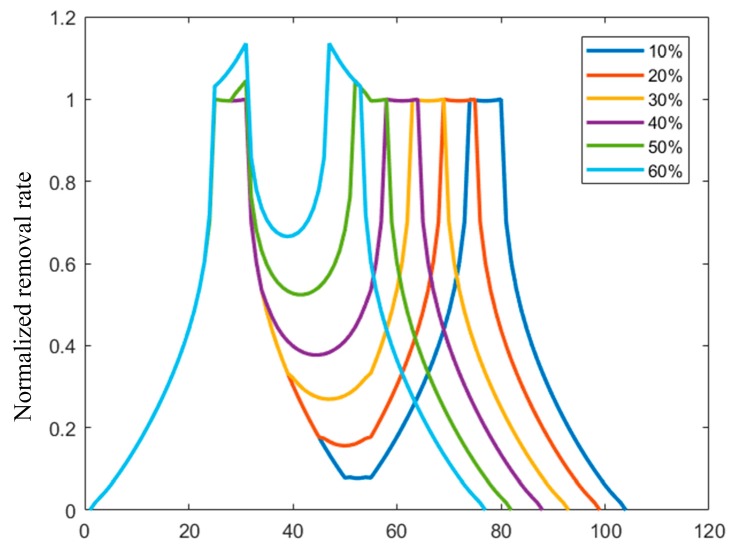
Cross-section diagram of the removal quantity of the polishing paths with different overlap rates.

The results are compared, as shown in Figure 8. The Y axis means the normalized removal rate. When the overlapping rate exceeded 40%, peaks of the removal rate emerged between the overlap of two paths; the peak on both ends will seriously influence the surface shape correction effect of the removal function. The same conclusion could also be obtained by analyzing the standard deviation. Thus, the 40% overlap rate will be selected to compensate for the surface profile error.

## 4. Strategies for Surface Waviness Compensation

**Figure 9:**
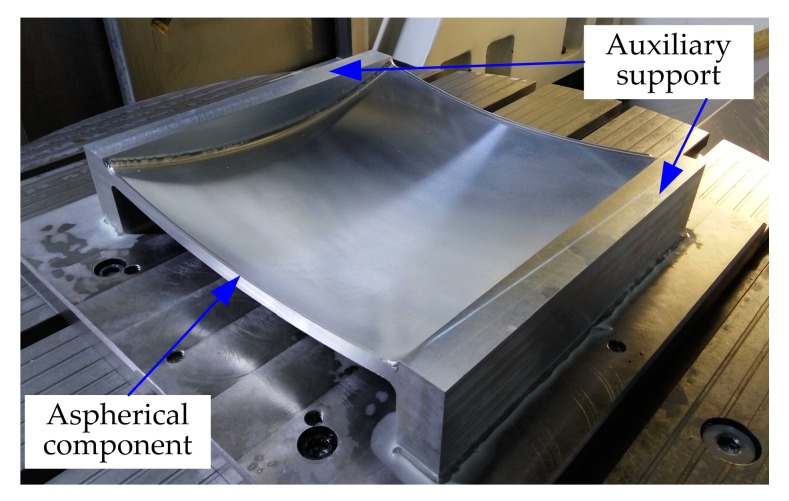
Machined aspherical component from the HSC 75 Linear.

**Figure 10:**
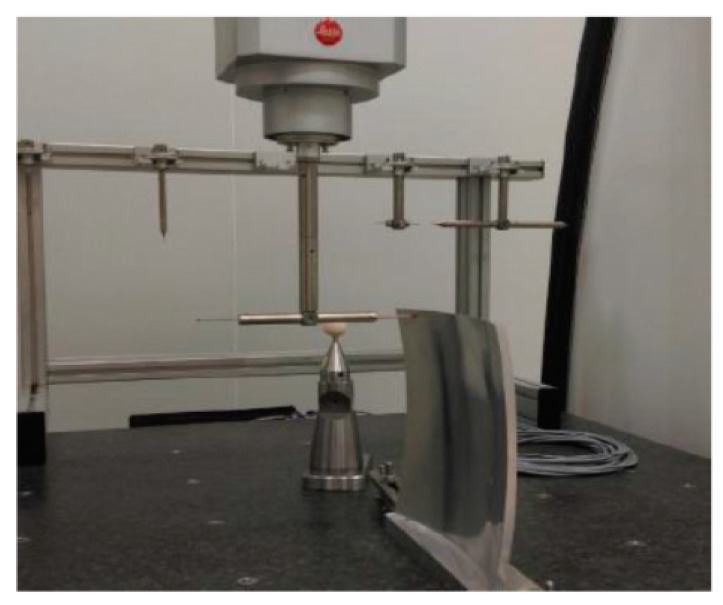
Equipment used in surface waviness measurement.

Different polishing paths were applied to compensate for the surface waviness. Before compensating for the waviness, all optical components were compensated for by the surface profile error by adopting the conventional raster path polishing method. Then, following the two strategies compensated for the waviness error. By comparing the results of the experiments, the application scenarios of the two methods were analyzed. In order to determine the performance of the two strategies, it is necessary to determine the evaluation method of surface waviness. Wavefront PSD is currently a reliable method for evaluating middle-frequency range errors of optical components [34]. The polishing equipment used in this study was a DMG five-axis CNC machining center HSC 75 Linear; the machined aspherical component is shown in Figure 9. The machining range along x, y, and z axes are 750, 600, and 560 mm, respectively. The rotational range of C-axis is 360° and the rotational range of B-axis is −10–110°. The measuring equipment was a coordinate measuring machine (CMM), as shown in Figure 10. The CMM (Infinity 12.10.7, Leitz, Oberkochen, Germany) with X/Y/Z has a measurement range of 1200/1000/700 mm, respectively, and a measurement error of 0.3 + L/1000 (μm) (L is the measurement length) in the entire workspace.

### 4.1. Changing Path Direction

**Figure 11:**
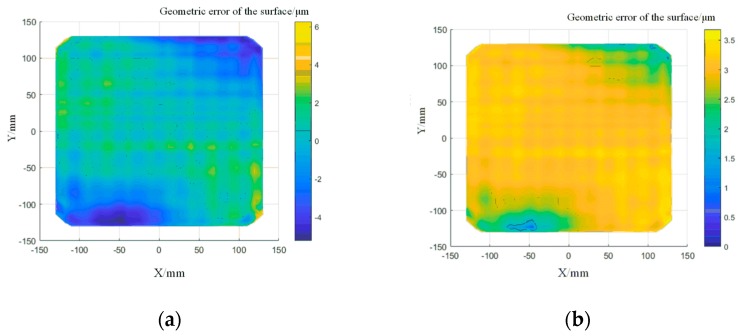
Profile error distribution after pseudo-random path polishing: (**a**) surface profile error distribution after ten times of polishing; (**b**) surface profile error distribution with pseudo-random path polishing.

A K9 optical glass (260 × 260 mm) was used in the experiment to verify the path direction changing algorithm. A semi-finished component, obtained by precision milling with a peak-valley (PV) value of 35.6 μm and root-mean-square (RMS) value of 15.47 μm, was used in the polishing process. The rotation speed of 1000 rpm and pressing depth of 1 cm were selected. Figure 11 shows the profile error distribution before and after the pseudo-random path polishing. The results show that the PV value converged to 3.58 μm and the RMS value converged to 1.06 μm. The figure suggests that the surface waviness error appears in the polishing process, but it could be reduced in order to obtain a relatively flat surface profile by applying the pseudo-random polishing path.

**Figure 12:**
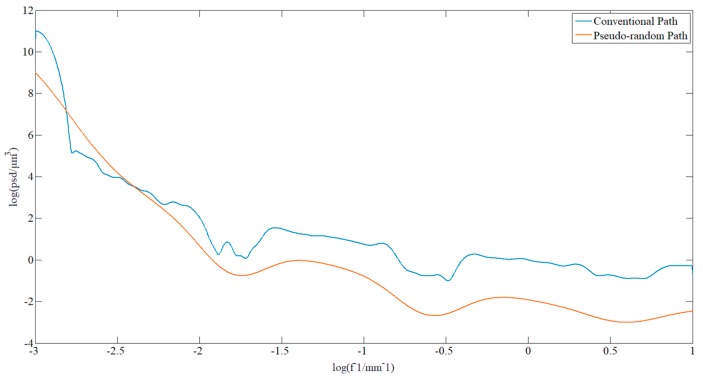
Comparison of the PSD of the sample before and after pseudo-random path polishing.

The pseudo-random path was applied in order to polish the component again. In order to determine the correct ability of the polishing path on the surface waviness, the surface shape error data was plotted into a PSD curve. The decrease in the curve indicates that the surface waviness has converged. The surfaces from the PSD data before and after the pseudo-random path polishing are shown in Figure 12. In the middle frequency range, the PSD curve decreased and peak-clipping was observed, which proved the validity of this method.

### 4.2. Changing Path Interval

**Figure 13:**
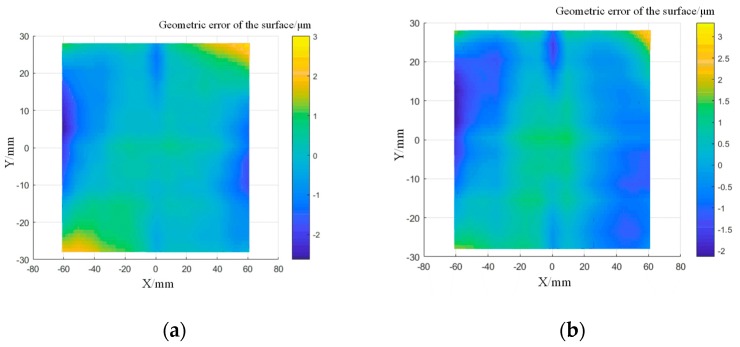
Distribution of the surface profile errors of the sample after compensation: (**a**) surface profile error distribution after five times of polishing; (**b**) surface profile error distribution after ten times of polishing.

A raster path based on the calculation of the overlap rate was applied to compensate for the surface profile error of the alumina component (120 × 55 mm). The polishing parameters are 1000 rpm rotation speed and 1 cm pressing depth. An overlap rate of 40% was set, according to the theoretical analysis described in the previous section. Figure 13 shows the error distribution after several surface profile corrections. The PV value and RMS value in the low frequency range of surface profile error were effectively reduced. The surface shape error converged to 5.3 μm and RMS to 1.56 μm.

**Figure 14:**
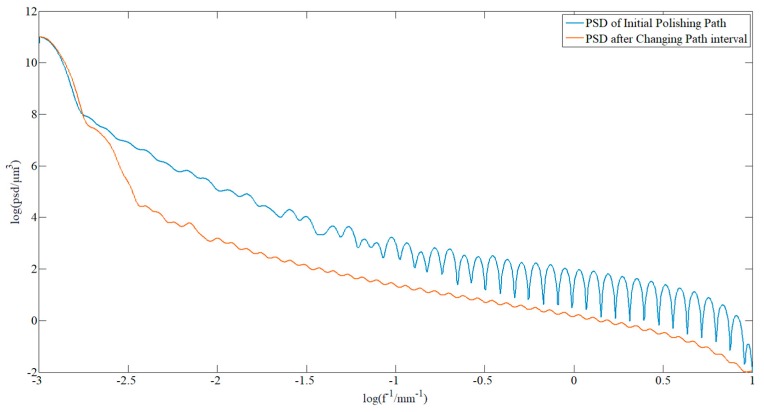
Comparison of the PSD curves of the sample before and after polishing with changing path intervals.

When processing the component, the strategy of changing the path interval was applied to facilitate a second dressing. Figure 14 shows the comparison of the PSD data before and after polishing. The middle part of the curve represents the surface waviness information, and it can be found that the correction effect was obvious in the middle frequency range, which means that the algorithm has a better correction effect on the surface waviness. However, the smoothness was worse than that from the pseudo-random path polishing, which illustrated that the method needs to be further improved.

## 5. Compensation of Surface Roughness

The strategy for improving surface roughness in the polishing process is to reduce the concentration of the polishing agent [35]. However, in order to ensure the correction efficiency of the surface profile, the polishing agent cannot be excessively diluted. Therefore, reasonable values are needed in order to balance the relationship between them. In this study, a 7075 series aluminum alloy was adopted as the experimental material, and aluminum oxide, with particle size of 1.5 μm, was selected as the polishing agent. Changes in the surface roughness with different concentrations of the polishing agents were evaluated through white light interferometry.

According to the concentration of the polishing agent and lapping oil, 10 types of polishing agents, with different concentrations, were configured. The surface roughness of the sample was Ra 12.5 before polishing. The surface of the material was polished for 5 min using different polishing agents. Table 2 shows the formulas of different polishing agents.

**Figure 15:**
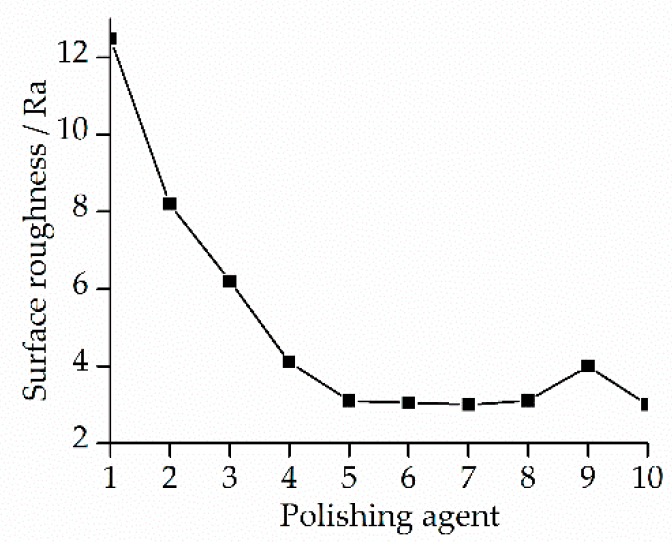
Roughness variation with different kinds of polishing agents.

Figure 15 shows the results of the 10 types of polishing agents. Ra was adopted in order to evaluate the surface roughness. The roughness became stable when the ratio of the lapping oil exceeded 50%. However, a scheme with a higher ratio of the lapping oil would greatly influence the error correction efficiency of the surface profile. Hence, based on the experimental conditions, the polishing agent with a ratio of 50% is far more appropriate, in comparison to the others.

**Figure 16:**
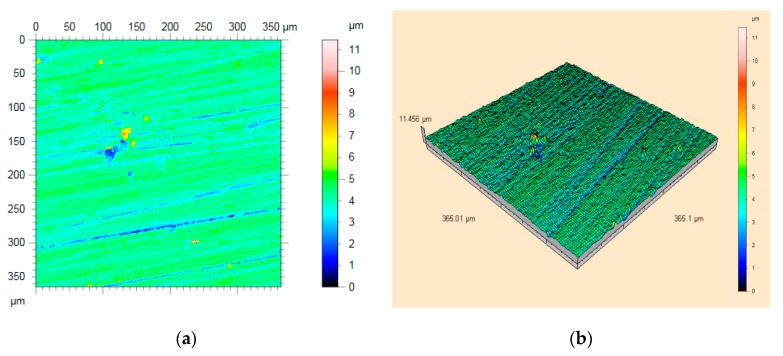
Surface roughness measurement results from white light interferometry: (**a**) a view of the surface topography; (**b**) three-dimensional roughness topography.

The roughness of all the surfaces were measured after polishing. Figure 16 shows the results from white light interferometer.

**Figure 17:**
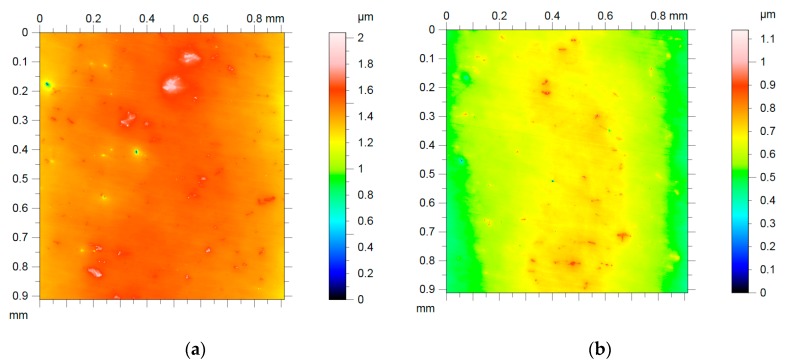
Surface roughness measurement for five times of polishing before and after replacement of the polishing agent: (**a**) before changing the polishing agent; (**b**) after changing the polishing agent.

The results of the white light interferometry are shown in Figure 17. The particle size of the alumina polisher used for surface compensation was W = 10 μm and W = 1.5 μm. The Ra value was 10.12 nm before using the new polishing agent, and it decreased to 2.03 nm after applying a 50% concentration of the polishing agent. Figure 16 shows the results of five times polishing before and after changing the polishing agent.

## 6. Conclusions

This study investigated the methods for improving the surface performance of optical components by modifying surface waviness and surface roughness, which correspond to middle and high frequency errors. Based on the experiments, the following conclusions can be drawn:

(1) The two path generation algorithms proposed in this paper are both feasible for correcting surface waviness. One problem caused by the existence of a large parallel path can be solved by the proposed path direction-changing algorithm, and another problem caused by the convolution effect between path intervals was solved by the path interval changing algorithm.
(2) The path generation algorithm that changes the path direction has better performance, but it has higher requirements on the performance of the machine tool. Therefore, it is suitable for optical components with higher precision requirements.
(3) Different path generation algorithms were applied to polish a workpiece made of K9 optical glass by a polishing agent concentration of 50%. The results show that the PV and RMS converge to 3.58 and 1.06 μm, respectively. Comparisons of the changes in PSD curves before and after polishing suggest that the two paths could correct surface waviness.

This method can be extended to the area of free-form precision polishing with a small grinding head. The surface error compensation for high-precision, large-diameter, non-rotationally symmetrical and free-form components would be the direction of our future work.

## Figures and Tables

**Table 1:** Standard deviation of the removal function with different overlap rates.

Overlap	Standard Deviation
10%	0.3057
20%	0.3030
30%	0.3015
40%	0.2933
50%	0.3258
60%	0.3679

**Table 2:** Formulas of different polishing agents.

Number	Polishing Agent (%)	Lapping Oil (%)
1	100	0
2	90	10
3	80	20
4	70	30
5	60	40
6	50	50
7	40	60
8	30	70
9	20	80
10	10	90
